# Electrochemical degradation of triclosan in aqueous solution. A study of the performance of an electro-Fenton reactor

**DOI:** 10.1016/j.jece.2019.103228

**Published:** 2019-08

**Authors:** Josué D. García-Espinoza, Irma Robles, Víctor Gil, Elías Becerril-Bravo, Jose A. Barrios, Luis A. Godínez

**Affiliations:** a Centro de Investigación y Desarrollo Tecnológico en Electroquímica S.C., Parque Tecnológico Qro. Sanfandila, 76703, Pedro Escobedo, QRO, Mexico; b Instituto de Ingeniería, Universidad Nacional Autónoma de México, 04510, CDMX, Mexico

**Keywords:** Water treatment, Electro-Fenton, Triclosan, Design of experiments, Advanced oxidation processes

## Abstract

The electro-Fenton degradation of Triclosan in aqueous solution was studied using a cylindrical reactor in which polarized carbon cloth electrodes and a cation exchange resin were employed. Using a factorial design of experiments approach, the effect of four variables (considering two levels for each one), was measured on four response parameters that reflect the electrooxidation efficiency of the electrochemical reactor. The results revealed that in all cases triclosan degradation was very efficient (above 95%) and that while there is a reasonable effect of all variables and their interactions, the one with the strongest influence on the process is the nature and magnitude of the ionic strength of the electrolytic solution. In this way, while the presence of a buffer species in this solution can keep the pH in a value that affects the generation of ^•^OH radicals from the Fenton mixture, a high ionic strength solution can promote the elimination of Fe ionic species from the reactor by decreasing resin Fe retention due to competition effects of other ions for the binding sites of the substrate. HPLC experiments of the effluent solutions, also revealed that the degradation by-products of triclosan were dependent on the nature and ionic strength of the electrolytic solution in the electro-Fenton process under study. Finally, comparison of the different operation modes, also suggested that electro-adsorption of Fe cationic species in the negatively polarized cathode surface, is the main factor that controls Fe ion retention within the reactor.

## Graphical abstract

**Figure:**
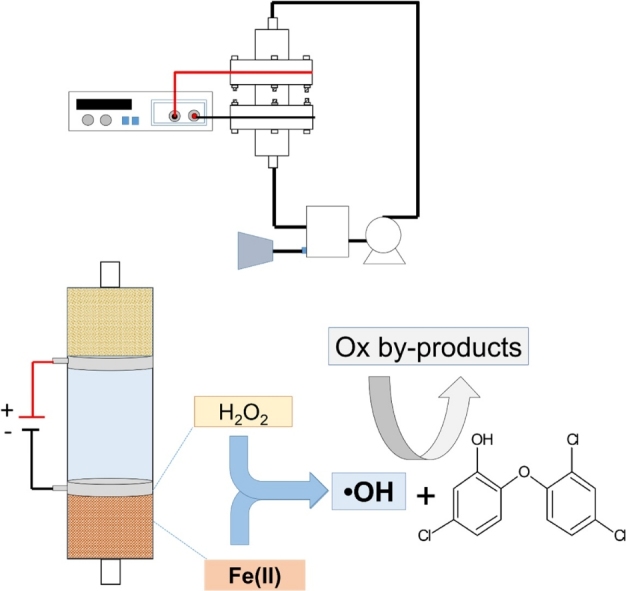

## 1 Introduction

In recent years, the presence of emerging contaminants in water sources that are dedicated to human consumption, has been detected and pointed out as an important health problem. Emerging pollutant species are not necessarily new contaminants; they could also be chemical compounds that have existed in the environment for a long time and just recently recognized as toxic or environmentally hazardous materials [1,2]. Typical emerging contaminants are components of some health care products, drugs, surfactants, plasticizers and a large group of additives that are frequently used in modern industry. Among these, triclosan is a popular chemical species that has been employed since the 70´s due to its ability to inhibit bacterial and fungi growth [3]. Triclosan has therefore been widely used in soaps and detergents and due to its low solubility, concentrations in the 0.5–1.3 μg/L range, have been found in samples of wastewater plants [4]. Recently, the presence of triclosan in urine of women that used a popular commercial toothpaste, has been reported [5].

Although biological processes are efficient for the removal of organic matter, their performance is limited when it comes to the degradation of chemically stable compounds such as triclosan. Along with other emerging contaminants, triclosan can be efficiently removed from aqueous effluents using the so-called Advanced Oxidation Processes (AOPs). These processes employ the ^•^OH radical species which, by virtue of its high oxidation potential (E° = 2.8 V *vs* reversible hydrogen electrode, RHE), is capable of degrading, and in some cases reaching full mineralization, of several persistent pollutants [6,7]. Their widespread employment however, has been limited due to factors associated to cost or difficulty, and good examples are electrochemical and photocatalytic oxidation processes. While in the first case expensive anode materials, such as boron doped diamond or dimensionally stable electrodes must be used [8,9], efficient photocatalytic materials capable of working with solar radiation, often need to be semiconductors doped with precious metals or rare earths [10,11].

In this context, efficient technologies based on affordable materials are being developed around the world. This is the case of the electro-Fenton process that employs carbonaceous electrodes which are characterized by low price and large surface area.

In this context, it is important to note that while there are different ways to generate the ^•^OH radical species, there are also reports on the use of different AOPs to remove triclosan from aqueous effluents. Among the AOPs explored for triclosan degradation, photocatalysis [12], Fenton [13], electro-Fenton [14], electrochemical oxidation [15] and ozonation [16], stand out as successful approaches and among these, the electro-Fenton treatment of contaminated water, is particularly attractive since it works by electrochemically producing H_2_O_2_ from dissolved oxygen reduction at a carbonaceous electrode surface (see Reaction (1)),

(1) $${\text{O}}_{2}+{2\text{H}}^{+}+{2\text{e}}^{-}\to {\text{H}}_{2}{\text{O}}_{2}$$

Combining this electro-generated species with Fe(II) ions in solution, ^•^OH radicals are readily produced as shown in Reaction (2).

(2) Fe2++H2O2→Fe3++ OH∙+OH-

The mixture of the two reactant species in Reaction (2) is commonly known as the Fenton reagent and as can be anticipated by examination of Eqs. (1) and (2), for a water treatment process based on this approach, it is necessary to produce and maintain the proper concentration of both species in solution.

In view of this limitation, several efforts have been made in order to overcome the intrinsic problems for the development of a feasible electro-Fenton water treatment technology. Among these, the efficient electrochemical production of H_2_O_2_, different releasing systems that make Fe(II) available for the ^•^OH producing reaction and the use of cation exchange resins that avoid the need to add acid and later neutralize it, have been explored in recent years by different research groups around the world [[17], [18], [19], [20]].

In the context of an electro-Fenton process that includes these considerations, the goal and novelty of this work, consist on the investigation and understanding of the influence, relative weight and interactions of the operational parameters under study, on the degradation performance of triclosan. In this regard, four operational parameters were chosen to assess their influence on triclosan removal, as well as in the concentration of Fe(II), pH and conductivity of the treated solution. These factors consisted on treatment time, amount of Fe(II), the nature and ionic strength of the electrolytic solution and the operation mode.

## 2 Experimental section

### 2.1 Materials

Analytical grade triclosan was obtained from Sigma-Aldrich. Methanol and formic acid as well as water and HPLC-grade acetonitrile were obtained from J.T. Baker. Membrane holders and 0.22 μm pore diameter membranes were obtained from Millipore and used to filter the samples. Na_2_SO_4_, HNO_3_, NaOH, FeSO_4_•7H_2_O and the compounds employed for the preparation of synthetic wastewater (SWW) [21] were also obtained from J.T. Baker. The commercially available cation exchange resin Amberlite was provided by Fluka (IR120) and carbon cloth was obtained from Grupo ROOE, Mexico.

Triclosan solutions were prepared with deionized water and the initial concentration of the pollutant species was set at 10 mg/L. Ultra-high performance liquid chromatography (UPLC) - mass spectrometry was employed for detection and quantification purposes, and while the mobile phase, A, was prepared by mixing 0.1% of formic acid in HPLC water, the mobile phase B, consisted on acetonitrile. Both phases were independently filtered through a 0.22 μm membrane before use. The two electrolytes employed in this study were prepared using deionized water and while the first one was obtained by dissolving Na_2_SO_4_ at a concentration of 0.05 M (ionic strength, I_Na2SO4_ = 1.5 × 10^−1^) ; the second one, a SWW solution (I_SWW_ = 2.441 × 10^−3^), was prepared according to Table 1 [21].

**Table 1:** Composition of synthetic wastewater.

Reactive	Concentration (mg/L)
Methanol	600
NH_4_Cl	90
K_2_HPO_4_	9
KH_2_PO_4_	8.4
FeSO_4_•7H_2_O	17.4
(NH_4_)6Mo_7_O_24_•4H_2_O	0.01
CaCl_2_•2H _2_O	4.4
MgSO_4_•7H_2_O	12.2
ZnSO_4_•7H_2_O	0.132
MnSO_4_•H_2_O	0.04
CoCl_2_•6H_2_O	0.03
EDTA	0.023

The cation exchange resin Amberlite on the other hand, was thoroughly washed with deionized water and then, partially exchanged with either FeSO_4_•7H_2_O or NaOH as previously reported [22,23]. The electrode carbon clothes were rinsed with deionized water, immersed in diluted HNO_3_ for 8 h and rinsed with deionized water.

### 2.2 Methods

**Fig. 1:**
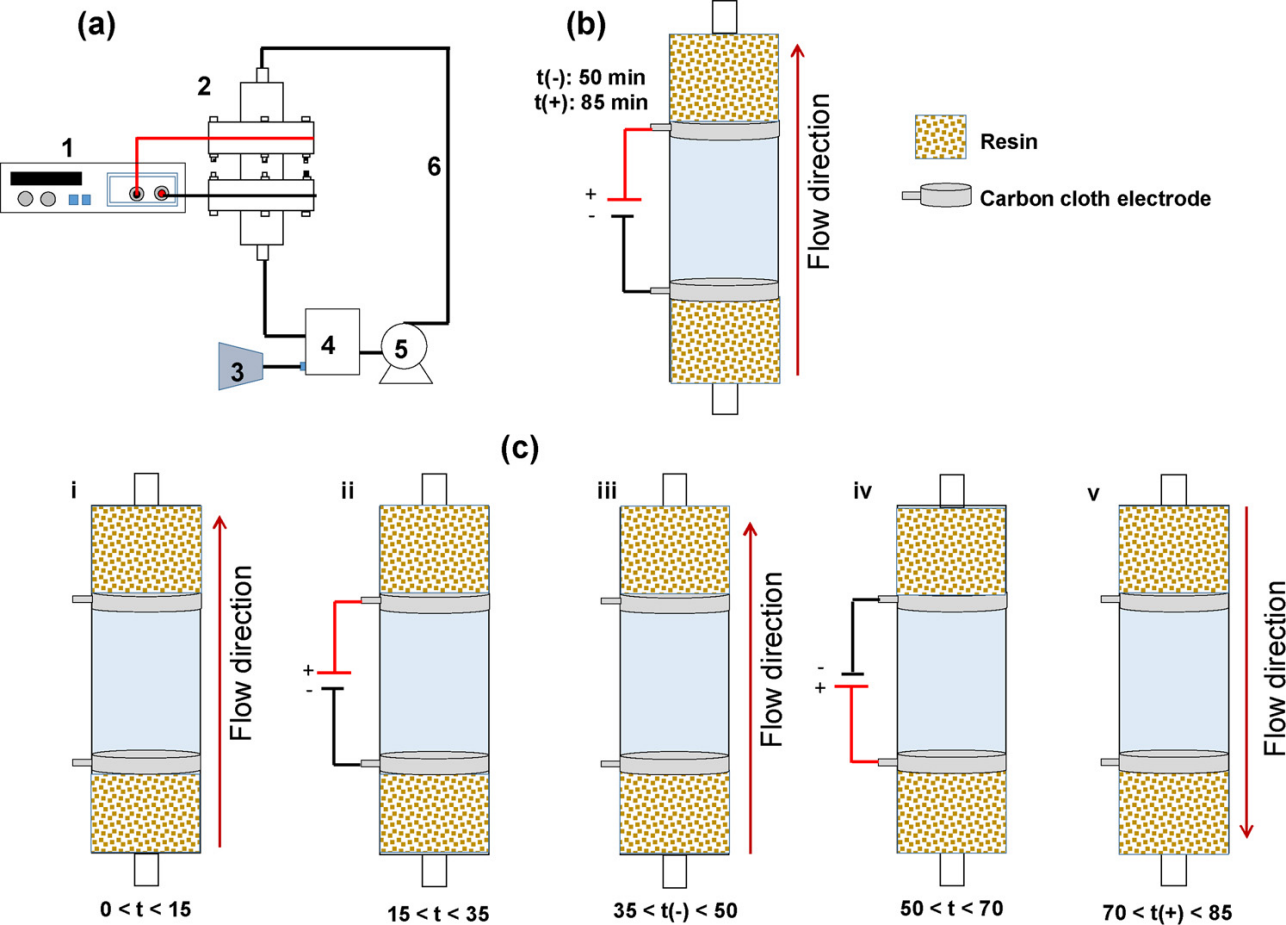
(a) Schematic diagram of the experimental set-up: (1) power source, (2) reactor, (3) air pump, (4) tank, (5) peristaltic pump, (6) recirculation pipe. Representation of (b) continuous and (c) batch (alternating polarization and flow direction) operation modes of the electro-Fenton process.

The electrochemical treatment of the contaminated aqueous solution, was carried out in a vertically oriented reactor, which, as can be seen in Fig. 1a, consists of three cylindrical pieces assembled in series: one central section made of polymethylmethacrylate with a volume of 30 mL, and two identical sections coupled on the up- and low-ends of the middle section using stainless steel screws. Carbon cloth circular pieces (4.91 cm^2^ effective area, 0.6 mm thickness, 0.5 Ω in^2^ electrical resistivity) served as electrodes positioned between the compartments with an inter-electrode gap of 6.5 cm. While in the compartment next to the cathode, a cation exchange resin containing a defined quantity of Fe(II) was placed, the same amount of a Na^+^-activated cation exchange resin was located in the section next to the anode. As can be seen in Fig. 1a, the reactor was fed in such a way that the triclosan solution passed through the three sections the reactor; that is, across the resin compartments and the polarized cloth-electrodes, flowing towards a receiving tank where the effluent solution was mixed with influent solution and from where the solution is pumped by means of a peristaltic device set at a flow rate of 30 mL/min. Oxygen on the other hand, was employed to continuously saturate the triclosan solution in the recirculation tank by means of an air pump. The working volume of the electrolytic solution was 0.2 L.

A potential difference between the two electrodes was applied by means of a power source in order to promote the 2e^−^ reduction of dissolved oxygen. The assessment of this potential difference, was performed from cyclic voltammetry experiments in which the potential *vs* a Ag|AgCl electrode for the 2e^−^ oxygen reduction reaction (ORR) was determined, and from potential measurements in the electrochemical reactor using the power source and a Ag|AgCl reference electrode positioned between the two carbon cloth electrodes. For these experiments, a BASi, potentiostat EC Epsilon and a Power Source (CIDETEQ) were employed.

The concentration of Fe(II) and total Fe, were determined following the methodology described by the APHA [24]. The Fe spectrophotometric measurements were carried out using a Genesys 10S, Thermo Scientific spectrophotometer. The pH and the conductivity of the solution on the other hand, were measured using a Thermo-Scientific potentiometer.

Triclosan concentration and degradation by-products were determined using an Acquity, ultra-high performance liquid chromatograph, UPLC class-H, Watters equipment, coupled to an Acquity QDa Watters mass spectrometer (QDa). A C18 Acquity UPLC Waters column (2.1 mm × 50 mm, 1.7 μm) and an injection volume of 20 μL were used in experiments performed at a flow of rate of 0.3 mL/min at 40 °C, with a mobile phase prepared with an A to B ratio of 30–70%. Chromatograms were obtained at 288 *m/z* in negative ionization mode and the QDa detector operated with a mass range between 100 and 300 *m/z*. The retention time of triclosan was 1.4 min and the total analysis time per sample was 3 min. Before every experimental determination, 2 mL of each sample was filtered through a 0.22 μm membrane.

The linear model obtained from the factorial design on the other hand, can be described using Eq. (3),

(3) Yj= β_0_+∑β_i_X_i_

Where *Y_j_* is the response variable, *X_i_* is the independent variable and *βo*, and *βi*, correspond to the constant and the linear model coefficient, respectively [25].

## 3 Results and discussion

### 3.1 Operational parameters for the factorial design study

In the proposed treatment process, the polluted solution passes through five stages that correspond to the different sections of the reactor: (i) the Fe(II) loaded resin, (ii) the negatively polarized carbon cloth, (iii) the electrolytic solution, (iv) the positively polarized carbon cloth and, (v), the Na^+^-loaded resin. In this way, in the first section of the reactor (i), the triclosan solution goes through and washes out Fe(II) ions from the resin material, taking them to section ii, where the carbon cloth simultaneously acts as an adsorbent and as a cathode in which oxygen reacts to produce H_2_O_2_. This species along with Fe ions, is the mixture known as the Fenton reagent which readily produces ^•^OH radical species at the electrode surface which, according to Eq. (2), imposes a strong oxidation environment that should decompose triclosan. After crossing section iii, the mixture of triclosan, its oxidation by-products and Fe cations, reach the second carbon cloth material, section iv, where additional oxidation reactions take place. Oxidation by-products along with Fe cationic species reach section v where, due to the cation exchange properties of the resin, the charged species are partially retained while letting pollutant-free effluent leave the reactor. It is important to point out that in this sequence, while ion exchange processes take place in the resin compartments (i and v), synergistic and simultaneous effects of adsorption–electrochemical degradation occur on both electrode surfaces (ii and iv).

As can be seen from this description and from inspection of Fig. 1b, when the treatment process is carried out in continuous mode, that is, maintaining both, the polarization in the electrodes as well as the flow direction constant, it is clear that eventually all the Fe cationic species will leave the reactor after being washed out from section v. In order to overcome this situation, a batch process can be defined in which the flow direction and the polarization of the two carbon cloths can be reversed in order to avoid the elimination of Fe from section v. Under this scenario, electrochemically generating Fenton conditions are kept within the reactor if the polarization and flow direction reverse step is carried out at an appropriate frequency [19]. As can be seen from Fig. 1c, a batch operation mode can therefore be carried out by defining sequential circulation and polarization stages for 15 and 20 min, respectively. Considering a first 15 min period of solution circulation so that the pollutant is adsorbed on the cathode electrode surface, one cycle was completed after 50 min and two cycles at 85 min. In these experiments, while the polarization stage was carried out under static solution conditions, in the case of the continuous operation mode, the solution flows steadily in the cathode to anode direction.

**Fig. 2:**
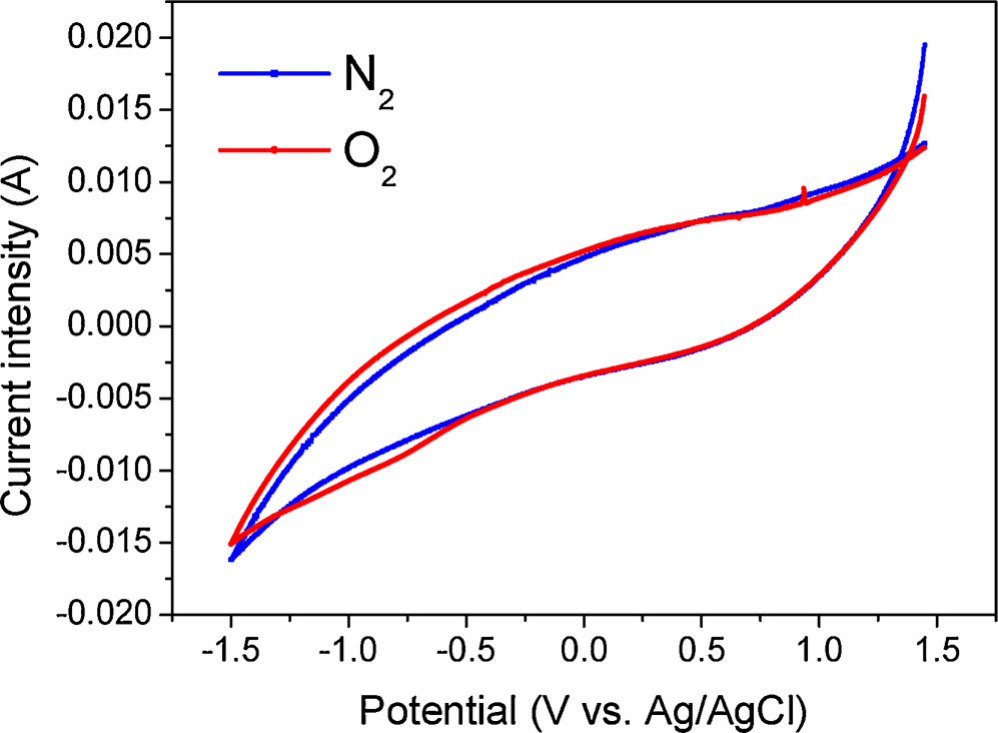
Cyclic voltammetry response of a 0.05 M Na_2_SO_4_ solution under nitrogen (blue line) and oxygen (red line) saturation conditions. Experiments carried out using carbon cloths as anode and cathode and Ag|AgCl as reference electrode. Scan rate 10 mV/s (For interpretation of the references to colour in this figure legend, the reader is referred to the web version of this article).

The polarization potential between the two electrodes in the reactor on the other hand, was determined from cyclic voltammetry (CV) measurements in which the potential for the 2e^−^ ORR at a carbon cloth electrode was measured. In this way, the identification of the corresponding reduction peak at the carbon cloth electrode was performed using an Ag|AgCl reference electrode by comparing the voltammetric response of N_2_ and O_2_ saturated, 0.05 M, Na_2_SO_4_ solutions (see Fig. 2).

Once this potential was identified (−0.8 V), the Ag|AgCl reference electrode was positioned between the two carbon cloth electrodes in the reactor, and with a power source, a polarization potential across the system was established in such a way that the cathodic carbon cloth electrode potential, corresponded to the ORR identified in the CV experiments.

It is important to point out that in addition to the operation mode (continuous *vs* batch) and reaction time, there are other important variables that is necessary to investigate since they are predicted to affect the performance of the electro-Fenton reactor under study. Consistent with previous reports [22,23], the amount of iron loaded resin as well as the ionic strength of the solution needed to be considered; particularly because these parameters affect the retention and distribution of the pollutant and the Fe ionic species across the reactor, and would therefore affect its performance in an important fashion.

In this context, four factors were chosen to build up the factorial design; and two levels were considered for each. As can be seen in Table 2, the resulting 2^4^ factorial design takes into account the treatment time (X_1_, which ranged between 50 and 85 min), the amount of Fe(II) loaded resin (X_2_), which varied between 0.5–1.0 g to complete 10 g of cation exchange resin (the remaining quantity as well as the resin in the opposite section of the reactor, consisted on Na^+^-activated resin), the nature and ionic strength of the electrolytic solution, (X_3_), which ranged between I_SWW_ = 2.441 × 10^−3^ for SWW and I_Na2SO4_ = 1.5 × 10^−1^ for the Na_2_SO_4_ solution, and the operation mode (X_4_); batch *vs* continuous.

**Table 2:** Experimental factorial design matrix.

Assay	Variables and levels				Variables			
	X_1_	X_2_	X_3_	X_4_	Time (min)	Amount of Fe(II) loaded resin (g)	Ionic strength	Operation mode
**1**	−1	−1	−1	−1	50	0.5	1.5 × 10^−1^	Continuous
**2**	+1	−1	−1	−1	85	0.5	1.5 × 10^−1^	Continuous
**3**	−1	+1	−1	−1	50	1	1.5 × 10^−1^	Continuous
**4**	+1	+1	−1	−1	85	1	1.5 × 10^−1^	Continuous
**5**	−1	−1	+1	−1	50	0.5	2.44 × 10^−3^	Continuous
**6**	+1	−1	+1	−1	85	0.5	2.44 × 10^−3^	Continuous
**7**	−1	+1	+1	−1	50	1	2.44 × 10^−3^	Continuous
**8**	+1	+1	+1	−1	85	1	2.44 × 10^−3^	Continuous
**9**	−1	−1	−1	+1	50	0.5	1.5 × 10^−1^	Batch
**10**	+1	−1	−1	+1	85	0.5	1.5 × 10^−1^	Batch
**11**	−1	+1	−1	+1	50	1	1.5 × 10^−1^	Batch
**12**	+1	+1	−1	+1	85	1	1.5 × 10^−1^	Batch
**13**	−1	−1	+1	+1	50	0.5	2.44 × 10^−3^	Batch
**14**	+1	−1	+1	+1	85	0.5	2.44 × 10^−3^	Batch
**15**	−1	+1	+1	+1	50	1	2.44 × 10^−3^	Batch
**16**	+1	+1	+1	+1	85	1	2.44 × 10^−3^	Batch

Table 2 also shows the 16 experiments which result from the 2^4^ factorial design using these variables. Considering one repetition for each combination, there are 32 experiments that were randomly carried out in order to avoid experimentalist bias.

### 3.2 Discussion of the experimental results of the factorial design

**Fig. 3:**
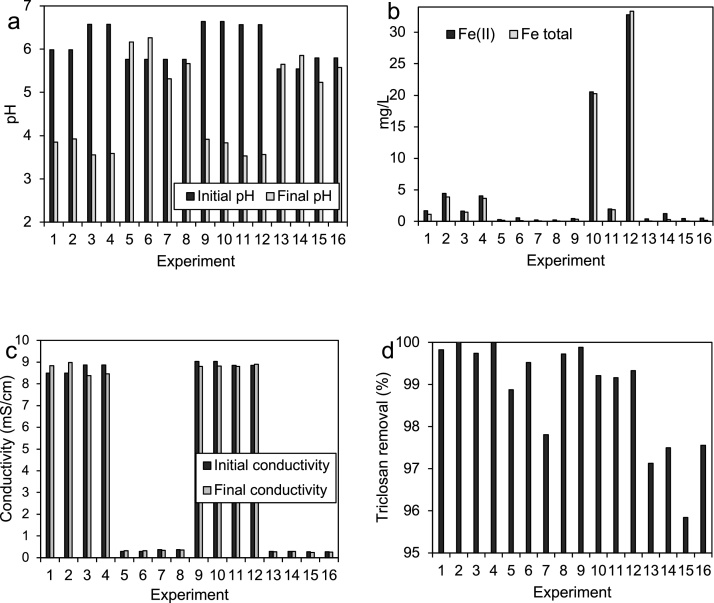
Values for the response variables under each experimental condition of the experiment design. (a) pH, (b) Fe(II) and total Fe, (c) conductivity and (d) triclosan removal percentage.

The results of the treatment for each combination of the factorial design, are shown in Fig. 3. Here, it can be readily seen that the response variables for analysis, consisted on the pH, the electric conductivity, and the concentration of Fe(II), total Fe and triclosan in the effluent.

Specifically, Fig. 3a shows the pH value of the aqueous solutions before and after the electro-Fenton treatment for all the process under study takes place. The data shows on one hand, that the initial pH value for all solutions is relatively close to neutrality; corresponding to 6.44 ± 0.42 and 5.71 ± 0.42 for Na_2_SO_4_ and SWW solutions, respectively. Inspection of the data after the electro-Fenton treatment is carried out, also shows that while there is an important decrease in pH for experiments 1–4 and 9–12, there is almost no change in the acidity level for the rest of the experimental runs. This acidity increase was expected from the interaction of the working solution with the acid groups of the resin and the surface of the carbon cloth electrodes. In this context, it is important to note that this effect was selective and that while the four runs that show a pH decrease (up to values of 3.71 ± 0.18) correspond to experiments that employ Na_2_SO_4_ as electrolyte (3.71 ± 0.18), the experiments for which the pH remains almost constant (5.71 ± 0.75) are those using the low ionic strength, SWW, as electrolyte. A reasonable explanation for this observation is related to the presence of KH_2_PO_4_ and K_2_HPO_4_ in the SWW electrolyte (see Table 1); a mixture of chemicals that act as a buffer agent in solution. In this context, it is also interesting to note that while a buffer effect in the electrolyte promotes neutral pH conditions in the effluent, thus making unnecessary a final neutralization stage for water discharge, the absence of this buffering effect favors mild acidic conditions in the electrolyte which, as it has been widely reported, constitutes a necessary condition for the efficient production of ^•^OH radicals from the Fenton mixture [6,7].

Another important factor to be considered for the process under study, consists on the concentration of Fe species in the effluent. There are two main reasons to try to minimize this concentration at the reactor’s outlet. While on one hand, the presence of Fe implies a separation step which carries an associated cost, its presence on the effluent also implies that this important component of the Fenton mixture is being lost from the reactor; thus imposing the need to monitor its concentration and replace the Fe ions that are lost in the process at the appropriate rate. The residual Fe concentrations in tank 4 in Fig. 1a for each experimental condition, are shown in the Fig. 3b. From the corresponding data, two observations stand out. While on one hand, there are some experiments for which the amount of Fe lost in the process is very high compared to the rest of the experiments, the differences between Fe(II) and total Fe values are very small for these particular tests; suggesting that the iron leaves the reactor mostly in its ferrous form. In this way, the experiments that showed the smallest Fe retention ability in the reactor (experiments 1–4 and 9–12), are those in which the Na_2_SO_4_ electrolyte was employed and therefore, those that are characterized by a high ionic strength of the electrolytic solution. A high concentration of ions in the electrolyte implies high competition for complexation sites in the resin and in the polarized electrodes; thus, inhibiting the retention of Fe ions in these sites, which in turn, favors their exit in the effluent. In the case of SWW, the opposite effect occurred, the low ionic strength allowed the Fe to interact with binding sites in the electrodes and resin, thus promoting higher retention of ionic iron (experiments 5–9 and 13–16).

Comparison of iron concentration values for different times in Fig. 3b reflect that as expected, experiments carried out for 85 min show in general terms, higher Fe leakage values than those obtained for experiments that lasted 50 min. Fe concentration comparison between operation modes however, also reveal an effect that is interesting and that confirms not only the important influence of the ionic strength, but also its relationship with electric polarization. In this way, as it can be seen by comparing the Fe concentration values of continuous *vs* batch modes for long time experiments (85 min) Fe leakage is unexpectedly larger for batch processes than for continuous operation assays (experiments 10 and 12 *vs* 2 and 4). Inspection of Fig. 3b reveals that at high ionic strength, polarization induced retention of Fe dominates over the cation exchange resin interaction; thus, showing that although continuous mode operation is characterized by longer circulation times (which should wash out Fe species), the longer polarization time favors Fe retention. This interpretation is also consistent with the fact that while differences between the amount of iron exchanged in the resin (0.5 and 1 g) do not seem to affect the amount of Fe lost in the continuous mode experiment (experiments 2 and 4), the difference is clearly reflected in the two batch mode processes surveyed (experiments 10 and 12).

As expected, and as can be seen in Fig. 3c, the ionic strength of the two electrolytic solutions studied did not change in an important fashion for any of the experiments employed. The conductivity values of 0.307 ± 0.05 mS/cm for SWW and 8.813 ± 0.35 mS/cm for Na_2_SO_4_, did not change due to the processes that take place in the reactor and, as it will be discussed in the following lines, did not affect in an important way the efficiency for triclosan degradation.

Triclosan degradation on the other hand, was very efficient in all the experimental conditions surveyed. As can be observed for the corresponding degradation percentage values in Fig. 3d, all the removal efficiencies were larger than 95%; and as expected, the larger values were obtained for the longest reaction times (even numbered experiments, see Table 2). Looking at the faster experiments (odd numbered experiments), it is interesting to note that three combinations (1, 3 and 9) stand out as highly efficient processes. These experiments have, as expected, different variable levels and show as a common feature the ionic strength and nature of the supporting electrolyte. In this way, these three experiments were carried out with solutions prepared with Na_2_SO_4_, a fact that not only conveys high ionic conductivity for the electrolytic solution, but also allows for mildly acidic conditions.

At his point, it is also interesting to compare the high triclosan removal percentage values obtained in this study, with those reported for different AOPs. Peng et al. [26] for example, reported that low concentration of graphene oxide increases the efficiency of a Fenton-like process, achieving 90% of triclosan degradation after 30 min (0.01 mM of Fe(III), 0.4 mM of H_2_O_2_, 0.02 mM of triclosan and 2.0 mg/L of graphene). On the other hand, complete degradation of triclosan and its by-products demanded 13.04 mg of ozone per mg of triclosan, following a pseudo-first order kinetics with an apparent reaction rate constant ranging from 0.214 min^−1^ to 0.964 min^−1^; depending on pH and initial triclosan concentration [16]. Liu et al. [27] also reported 78.7% of triclosan degradation within 30 min by photo-electrocatalysis using Na_2_SO_4_ as electrolyte and Azarpira et al. [28] evaluated the photocatalytic removal of triclosan with UV/iodine/ZnO. In this report, the authors found that after 20 min of reaction, the degradation efficiency of UV-only, UV/ZnO, UV/Iodide and UV/iodine/ZnO processes were 12.3, 37.3, 50.43 and 89.83%, respectively.

### 3.3 Variable effect analysis

Using the factorial design shown on Table 2 and the resulting experimental data on Fig. 3, empirical relationships for each response, *Y_j_*, and the variables, *X_i_*, under study, were calculated as described in Eq. (3). The resulting polynomial Eqs. (4), (5), (6), (7), (8), show up to second order interaction terms, that have different contribution effects on the corresponding response variable.

(4) $${\text{Y}}_{\text{T}\text{r}\text{i}\text{c}\text{l}\text{o}\text{s}\text{a}\text{n}}=98.91+0.29{\text{X}}_{1}-0.16{\text{X}}_{2}-0.82{\text{X}}_{3}-0.63{\text{X}}_{4}+0.22{\text{X}}_{1}{\text{X}}_{2}+0.29{\text{X}}_{1}{\text{X}}_{3}-0.088{\text{X}}_{1}{\text{X}}_{4}-0.097{\text{X}}_{2}{\text{X}}_{3}-0.045{\text{X}}_{2}{\text{X}}_{4}+0.36{\text{X}}_{3}{\text{X}}_{4}$$

(5) $${\text{Y}}_{\text{F}\text{e}(\text{I}\text{I})}=4.45+3.58{\text{X}}_{1}+0.77{\text{X}}_{2}-3.98{\text{X}}_{3}-2.82{\text{X}}_{4}+0.58{\text{X}}_{1}{\text{X}}_{2}-3.44{\text{X}}_{1}{\text{X}}_{3}-2.99{\text{X}}_{1}{\text{X}}_{4}-0.90{\text{X}}_{2}{\text{X}}_{3}-0.87{\text{X}}_{2}{\text{X}}_{4}+2.66{\text{X}}_{3}{\text{X}}_{4}$$

(6) $${\text{Y}}_{\text{t}\text{o}\text{t}\text{a}\text{l}\;\text{F}\text{e}}=4.17+3.54{\text{X}}_{1}+0.91{\text{X}}_{2}-4.05{\text{X}}_{3}-2.87{\text{X}}_{4}+0.68{\text{X}}_{1}{\text{X}}_{2}-3.50{\text{X}}_{3}-2.93{\text{X}}_{1}{\text{X}}_{4}-0.94{\text{X}}_{2}{\text{X}}_{3}-0.91{\text{X}}_{2}{\text{X}}_{4}+2.84{\text{X}}_{3}{\text{X}}_{4}$$

(7) $${\text{Y}}_{\text{p}\text{H}}=4.72+0.066{\text{X}}_{1}-0.21{\text{X}}_{2}+1.00{\text{X}}_{3}+0.073{\text{X}}_{4}+0.030{\text{X}}_{1}{\text{X}}_{2}+0.0{59\text{X}}_{1}{\text{X}}_{3}+0.0039{\text{X}}_{1}{\text{X}}_{4}-0.053{\text{X}}_{2}{\text{X}}_{3}-0.046{\text{X}}_{2}{\text{X}}_{4}+0.062{\text{X}}_{3}{\text{X}}_{4}$$

(8) $${\text{Y}}_{\text{C}\text{o}\text{n}\text{d}.}=4.53+0.023{\text{X}}_{1}-0.058{\text{X}}_{2}-4.22{\text{X}}_{3}+0.025{\text{X}}_{4}+0.0017{\text{X}}_{1}{\text{X}}_{2}-0.017{\text{X}}_{1}{\text{X}}_{3}+0.0075{\text{X}}_{1}{\text{X}}_{4}+0.052{\text{X}}_{2}{\text{X}}_{3}-0.057{\text{X}}_{2}{\text{X}}_{4}+0.062{\text{X}}_{3}{\text{X}}_{4}$$

In order to assess the percentage effect of each variable and the corresponding interactions on a particular response, Eq. (9) was employed [29].

(9) $${\text{P}}_{\text{i}}=\left(\frac{{\text{b}}_{\text{i}}^{2}}{\sum_{\text{i}=1}^{\text{k}}{\text{b}}_{\text{i}}^{2}}\right)100\;\left(\text{i}\neq 0\right)$$

In this equation, *P_i_* is the percentage contribution of each independent variable *X_i_*, and *b_i_* represents the estimation of the main effect of each variable.

**Fig. 4:**
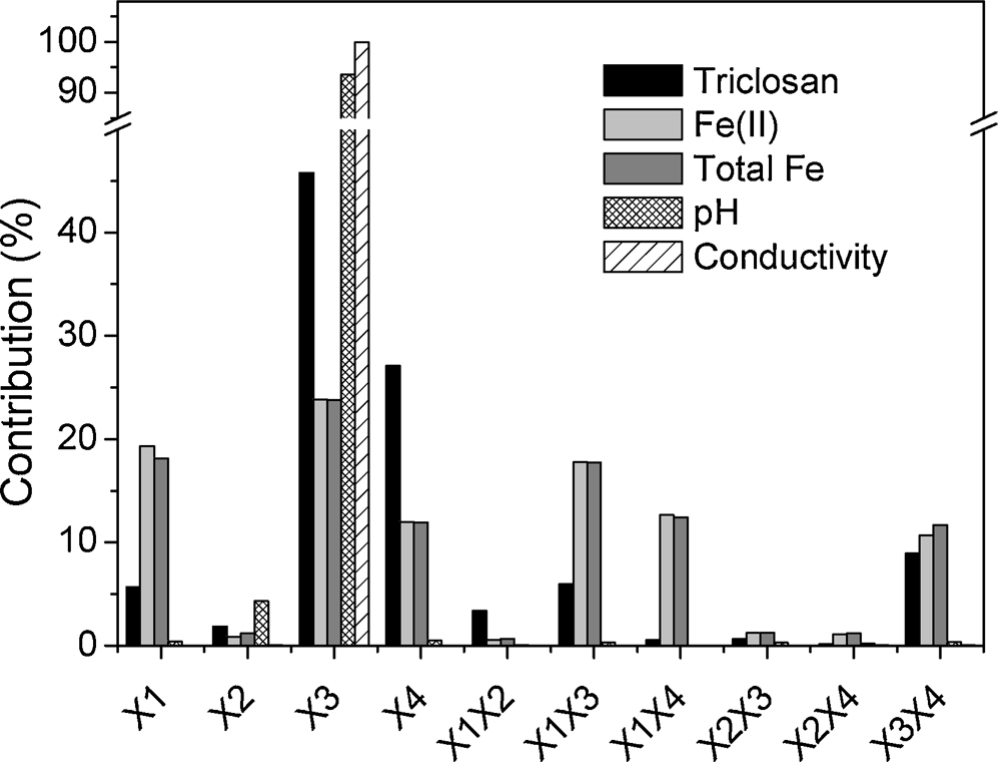
Standardized factor contributions for the degradation of triclosan, Fe(II) and total Fe concentration, pH and conductivity (X_1_: treatment time; X_2_: resin with Fe(II); X_3_: ionic strength; X_4_: operation mode).

Fig. 4 shows the percentage contribution of each factor in the responses surveyed. The contributions of the independent factors in the removal of triclosan were 45.81% for the ionic strength (X_3_), 27.08% for the operation mode (X_4_), 5.66% for the treatment time (X_1_) and 1.85% for the amount of Fe(II) resin (X_2_). In a similar fashion, ionic strength has an influence of 23.84 and 23.77% on the remnant concentration of Fe(II) and total Fe, respectively; reaction time impacts 19.31 and 18.12%; the operation mode 11.98 and 11.94% and the amount of resin, 0.89 and 1.19 for Fe(II) and total Fe, respectively. On the other hand, for both, the pH value of the effluent and in its conductivity, the predominant variable is once again the ionic strength, with a contribution of 93.54 and 99.9%, respectively. Regarding the factors that describe the effect of variable interactions, the data shows that while X_1_X_3_ has an effect of 17.78 and 17.73% in the concentration of Fe(II) and total Fe, respectively, interaction X_1_X_4_ showed 12.63 and 12.41% for the same response parameters. The data shown in Fig. 4, also reveals that the other interactions influence the responses less than 11%. In the light of these results, it can be clearly seen that the ionic strength is the pivotal variable, mainly for the removal of triclosan, pH and conductivity of the treated water, and to a lesser extent for Fe(II) and total Fe.

The correlation factor, R^2^, is the proportion of the variability in the response variables, which is accounted by a regression analysis and it is a measure of the degree, or quality, of the fitting process. In this way, for a good fit, R^2^ should be at least 0.80 [30]. The R^2^ value obtained for the data from the experiments described in this work were 0.8324, 0.8323, 0.8326, 0.8310 and 0.9987 for the removal of triclosan, Fe(II), total Fe, pH and conductivity, respectively. Since these values are all larger than 0.80, it can be concluded that, within the considered ranges, the models obtained describe reasonably well the behavior of the electro-Fenton process under study.

### 3.4 By-products investigation

This investigation suggested that among the factors considered, the nature and concentration of the supporting electrolyte is the most influential parameter for the performance of the electro-Fenton process studied. In this regard, and although high degradation efficiencies were obtained for all combinations, we wondered if the electrolyte nature and concentration would also affect the actual degradation path of triclosan. For this purpose, *m/z* spectra for each experimental combination surveyed was obtained as described in the experimental section. The resulting data (see Table 3), shows that as expected, the two electrolytes employed define two groups of by-products of triclosan degradation.

**Table 3:** *m/z* of the generated by-products at 1.419 min of retention time.

Assay	*m/z*
1	164.85, (164.91), 216.93, (216.93), 238.91, (238.91), 286.95, (286.86)
2	164.86, (164.86), 194.98, (194.98), 216.89, (216.89)
3	164.9, (164.92), 216.86, (216.93), 238.88, (238.9)
4	164.86, (164.9), 216.88 (215.92), 238.92, (259.86)
5	178.95, (195.79), 197.78 (197.76), 206.97, (286.93), (288.92)
6	178.93, (195.81), (197.76), (286.91)
7	178.91, (195.82), 197.83, (197.78), 206.98, (286.92), (288.99)
8	178.92, 195.79, 197.8, (197.8), 206.97, (286.91)
9	216.93, (216.93), 259.86, (259.86)
10	164.87, (164.84), 216.89, (216.91), 238.91, (238.93), 286.89, (286.99), (288.99)
11	164.9, (164.9), (194.94), 216.83, (216.95), 286.92, (286.96)
12	164.88, (164.89), (194.91), 216.96, (216.88), (238.99), 286.94, (286.9), 288.96, (288.93).
13	195.78, (195.74), 197.8, (197.77), 199.8, 286.98, (286.94)
14	195.81, (195.8), 197.78, (197.84), (286.95)
15	195.77, (195.79), 197.79, (197.8), (286.9)
16	195.79, (195.7), 197.79, (197.81), 286.96, (286.95), (288.98)

(The values in the parenthesis are the *m/z* recorded in duplicate experiments).

In this way, experiments 1–4 and 9–12, *i.e.*, those carried out in the presence of Na_2_SO_4_ as electrolyte, have a *m/z* signal from triclosan degradation by-products at 164 *m/z.* This signal can be indexed as 2,4-dichlorophenol, which probably results from the hydroxylation reaction on the C(1)- or C(1´)-position of triclosan [14]. Under these high ionic strength conditions, there is also an intermediate with an *m/z* of 217 which suggests the loss of two chlorine atoms [31].

At low ionic strength on the other hand, dichlorohydroquinone (178 *m/z*) was identified, which probably results from ^•^OH attack on the C(4)-position of 2,4-dichlorophenol with the coupled loss of the Cl^−^ ion. Since 2,4-dichlorophenol was only detected at high ionic strength, and since this is the precursor of dichlorohydroquinone, it seems reasonable to postulate that, under these conditions, its fast formation and transformation takes place. In addition, *m/z* of 198 suggests the generation of trichlorophenol or the loss of three chlorine atoms and the coupled introduction of a hydroxyl group.

**Fig. 5:**
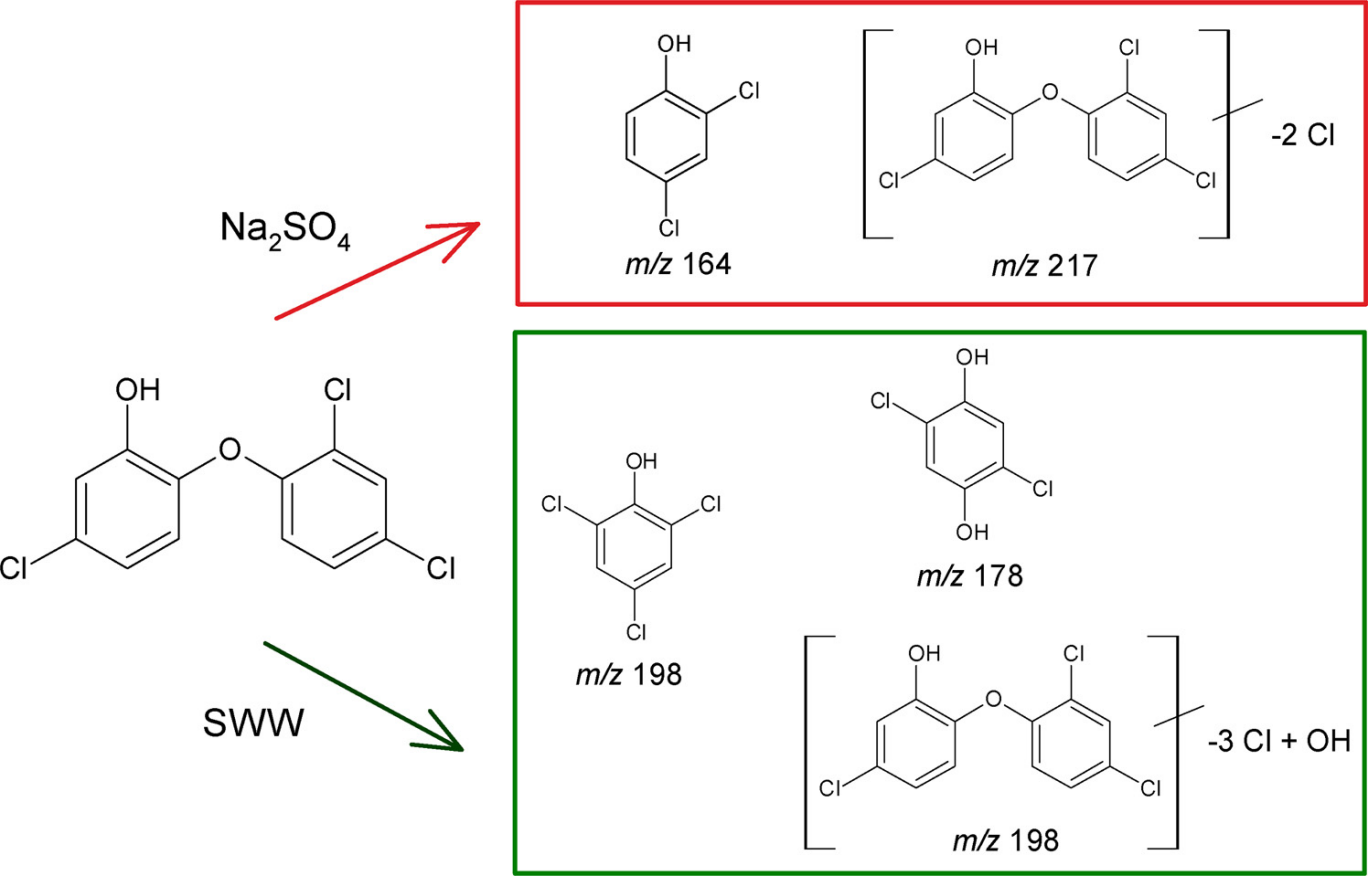
Degradation pathway of triclosan by the electro-Fenton process under study in Na_2_SO_4_ and SWW electrolytes.

Finally, the *m/z* of 288, which corresponds to the remnant triclosan species, appeared mostly in high ionic strength environment, a fact that is in agreement with the slightly low triclosan degradation efficiency found at low ionic strength. Based on the identified intermediates, the degradation scheme proposed is shown in Fig. 5.

It is also important to point out that some triclosan degradation intermediates are more toxic and stable than their parent compound. This is the case for 2,7-dichlorodibenzodioxin [32]. However, the reported *Vibrio fischeri* acute toxicity of 2,4-dichlorophenol (EC_50_, half-maximal effective concentration = 4.9 mg/L) is almost thirteen times lower than that of triclosan (EC_50_ = 0.39 mg/L) [13,32]. At higher trophic level, using *Danio rerio* organism, Zhang et al. [33] reported a LC_50_ (lethal concentration) and EC_50_ values of 0.51, 1.11, 2.45 mg/L, and 0.36, 0.74, 1.53 mg/L for triclosan, 2,4,6- trichlorophenol and 2,4- dichlorophenol, respectively. The obtained results are therefore consistent with the ones reported by García-Espinoza and Mijaylova Nacheva [34], in which the composition of the electrolyte controls the removal efficiency of three pharmaceuticals as well the generated by-products and the toxicity of the treated solution.

It is important to note that the identified intermediates may be further degraded by ring-rupturing reactions, yielding more simple compounds, which through further oxidation could be mineralized. Nonetheless, further assays need to be addressed to evaluate the potential mineralization of the electro-Fenton process.

## 4 Conclusions

In this work, four operational parameters were studied in terms of their influence on the same number of response variables which, from an integral perspective, reflect the performance of an electro-Fenton reactor for the degradation of triclosan in aqueous solution. The effect of these variables on the reactor´s performance was measured, discussed and fitted to empirical equations. From the experimental data, the individual and combined effects of the nature and ionic strength of the electrolytic solution, probed to be the most important variable of the process. In this way, while the presence of buffer species affects the pH and the efficiency of ^•^OH radical generation from the Fenton mixture, high ionic concentration implies less capacity of the resin for Fe cation retention within the reactor and therefore, less efficiency of the electro-Fenton process.

In any case, this study only correlates the chosen variables bounded by the level limits of the experimental design. Studies of this type of electrochemical advanced oxidation processes with complex samples and real wastewater solutions, is necessary to test these findings and further advance the understanding of the relationship between the operational and response variables of an electro-Fenton reactor.
